# Supplementary material for: Evaluation of Cognitive Functions in People Living with HIV Before and After COVID-19 Infection
Source: Viruses. 2025 Jan 20;17(1):135. doi: 10.3390/v17010135 (PMC11769327; doi:10.3390/v17010135)
Supplement: Supplementary file 1 [file viruses-17-00135-s001.zip › viruses-3405785-Figure S1.pdf]

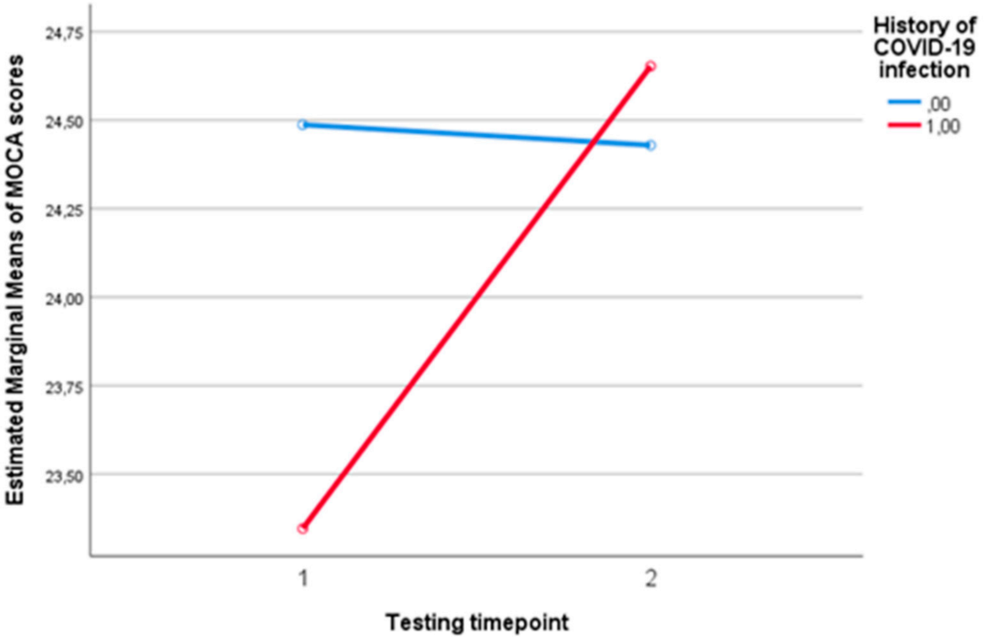

Figure S1. Estimated marginal means of MOCA scores over two timepoints for individuals with and without a history of COVID-19 infection. Covariates appearing in the model are evaluated at the following values: age at second testing = 49.8594, anxiety score = 4.6167, depression score = 3.7833. No significant differences were observed over time, between groups or in the interaction between time and COVID-19 history (all $p > 0.05$). MOCA: Montreal Cognitive Assessment, COVID-19: coronavirus disease 2019.
